# Effects of tigecycline and doxycycline in porcine endotoxemia

**DOI:** 10.1186/cc9656

**Published:** 2011-03-11

**Authors:** M Von Seth, J Sjölin, A Larsson, M Eriksson, M Lipcsey

**Affiliations:** 1 Department of Surgical Sciences, Uppsala University, Uppsala, Sweden; 2 Department of Medical Sciences, Uppsala University, Uppsala, Sweden

## Introduction

Tigecycline, the first drug in a new class of antibiotics, the glycylcyclines, is used in the treatment of severe abdominal and connective tissue infections. Tetracyclines, having a structure-activity relationship with tigecycline, exert anti-inflammatory effects [1]. Some laboratory studies suggest that tigecycline may have anti-inflammatory properties in sepsis, but this has not previously been explored in a large animal integrative intensive care model.

## Methods

Eighteen piglets weighting 25.0 ± 2.2 (mean ± SD) were randomized to receive tigecycline 100 mg, doxycycline 200 mg or placebo and subjected to 6 hours of endotoxin infusion of 2 μg/kg/hour. We measured inflammatory, hemodynamic and respiratory variables.

## Results

TNFα was lower in the doxycycline group compared with the tigecycline and placebo groups during the experiment 0 to 6 hours (*P *< 0.05). The mean arterial pressure decline from baseline was greater during the experiment in the placebo group compared with the tigecycline group 0 to 6 hours (*P *< 0.05), but not the doxycycline group.

## Conclusions

Doxycycline demonstrated anti-inflammatory properties. Tigecycline counteracted emerging circulatory deterioration without affecting the proinflammatory cytokine response in this model.
